# A shape-adjusted ellipse approach corrects for varied axonal dispersion angles and myelination in primate nerve roots

**DOI:** 10.1038/s41598-021-82575-9

**Published:** 2021-02-04

**Authors:** Petra M. Bartmeyer, Natalia P. Biscola, Leif A. Havton

**Affiliations:** 1 Department of Neurology, David Geffen School of Medicine at UCLA, Los Angeles, CA USA; 2 School of Electrical and Computer Engineering at University of Campinas, Campinas, SP Brazil; 3 Department of Neurology, Icahn School of Medicine at Mount Sinai, New York, NY 10029 USA; 4 Departments of Neurology and Neurobiology, David Geffen School of Medicine at UCLA, Los Angeles, CA USA; 5 Departments of Neurology and Neuroscience, Icahn School of Medicine at Mount Sinai, New York, NY USA; 6 Neurology Service and RR&D National Center for the Medical Consequences of Spinal Cord Injury, James J. Peters Veterans Administration Medical Center, Bronx, NY USA

**Keywords:** Neuroscience, Peripheral nervous system, Autonomic nervous system

## Abstract

Segmentation of axons in light and electron micrographs allows for quantitative high-resolution analysis of nervous tissues, but varied axonal dispersion angles result in over-estimates of fiber sizes. To overcome this technical challenge, we developed a novel shape-adjusted ellipse (SAE) determination of axonal size and myelination as an all-inclusive and non-biased tool to correct for oblique nerve fiber presentations. Our new resource was validated by light and electron microscopy against traditional methods of determining nerve fiber size and myelination in rhesus macaques as a model system. We performed detailed segmental mapping and characterized the morphological signatures of autonomic and motor fibers in primate lumbosacral ventral roots (VRs). An *en bloc* inter-subject variability for the preganglionic parasympathetic fibers within the L7-S2 VRs was determined. The SAE approach allows for morphological ground truth data collection and assignment of individual axons to functional phenotypes with direct implications for fiber mapping and neuromodulation studies.

## Introduction

Significant advances in light and electron microscopy, using novel computer-assisted and automated image capturing and tiling, digital segmentation, and quantitative studies of nerve fiber size and myelination, have markedly increased the speed of data analysis and throughput, making possible new insights into the fine organization of the nervous system at high resolution^1–4^. For instance, recent 3-dimensional ultrastructural studies and diffusion magnetic resonance imaging (MRI), have suggested caliber fluctuations along myelinated axons and a non-linear orientation for many myelinated fibers in nerve bundles^3,5^.

Theoretical and applied studies on the electrical, morphological, and conduction properties of myelinated axons generally regard nerve fibers as cylindrical structures with a circular cross section^6–15^. However, these well-established methods for determining nerve fiber size do not take into consideration atypical nerve fiber shapes or the dispersion angle formed between each individual fiber segment’s direction and the mean direction of the overall nerve bundle. Therefore, fibers sectioned at oblique planes present with an enlarged cross-sectional area, and their size and degree of myelination are subsequently overestimated, when traditional morphometric approaches are applied.

Accurate measurements of fiber size and myelination are critical for determining physiologic phenotypes and developing improved theoretical neuromodulation models. Individual axons within a peripheral nerve show a wide range of anatomical and physiological features, and each fiber may be classified into a distinct physiologic type taking into consideration e.g. its axonal caliber, conduction velocity, and degree of myelination or G-ratio^16–20^.

Here, we developed a novel approach for the correction of size and myelination of individual nerve fibers for light and electron microscopy, taking into consideration the shape and dispersion angle for individual fibers, in a rhesus macaque model system. The L6–S3 ventral roots (VRs) were selected as lumbosacral VRs carry large and medium-sized α- and γ-motor fibers, respectively, and some VRs also carry small preganglionic parasympathetic fibers, all readily identified based on size, myelination, and conduction properties^21–24^.

## Results

**Figure 1:**
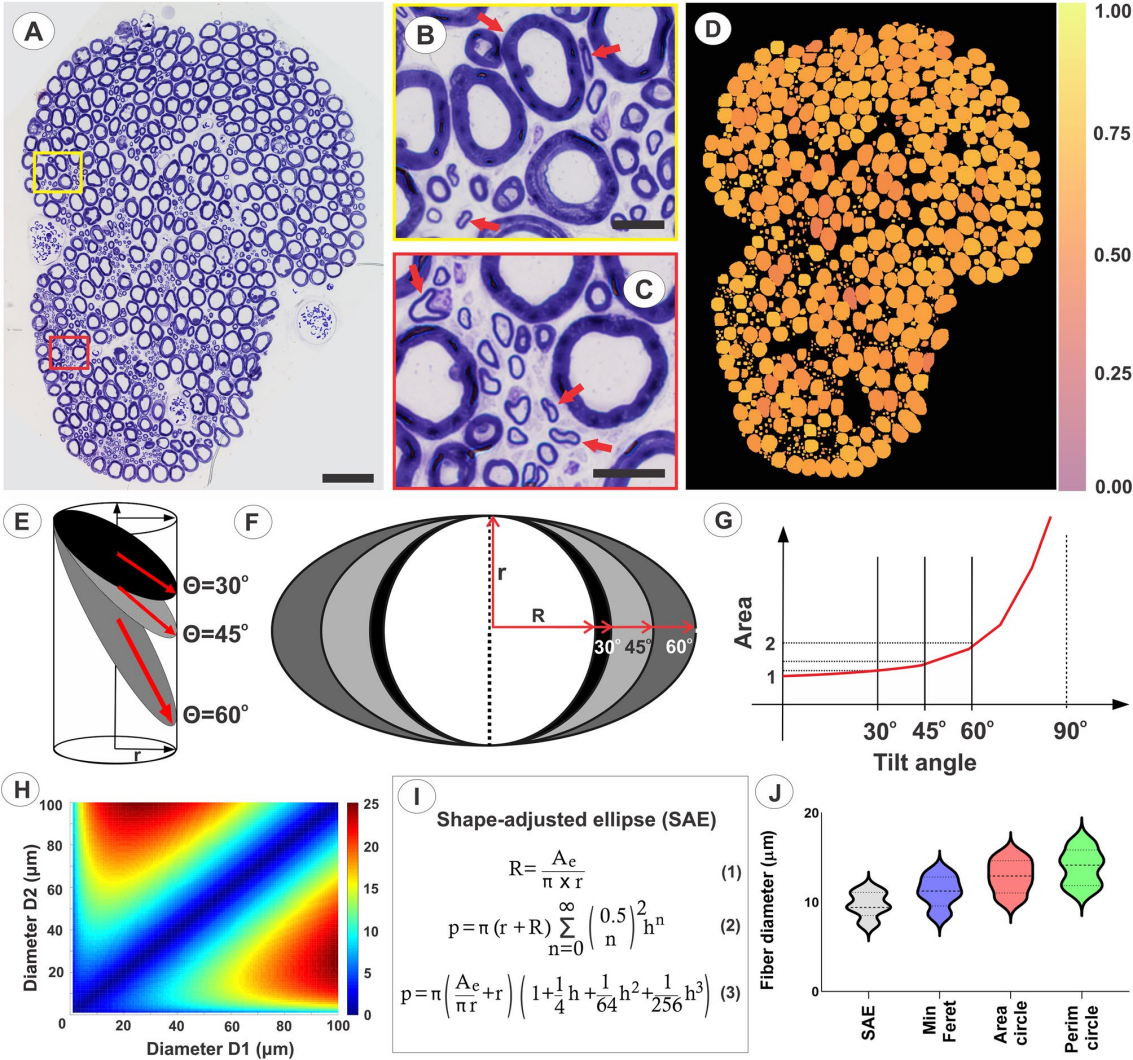
Shape adjusted ellipse (SAE) determination of nerve fiber size. (**A**) Transverse section of toluidine blue stained S1 ventral root in a female rhesus macaque. Note cross-sections of myelinated fibers of varied sizes. (**B,C**) Close-up view of boxed areas in (**A**), showing fiber shape heterogeneity, including presence of several elongated fiber presentations. Arrows indicate examples of non-circular shapes. (**D**) Heat map for S1 ventral root in (**A**) showing calculated roundness of myelinated fibers as a representative indicator for shape. Note marked heterogeneity of roundness for fibers across all sizes. A calculated roundness of 1 indicates a circular shape. (**E**) Sectioning of a cylinder with a circular base sectioned at different angles results in ellipse-shaped presentations of cut surfaces. (**F**) A cylinder in (**E**) sectioned at increasing tilt angles shows increased elongation and length of the long diameter of the corresponding ellipse-shaped cut surface. Note that the short diameter of the ellipse remains unchanged at all tilt angles. (**G**) Graph depicting error with overestimation of cylinder diameter, when the formula for a circle is used to calculate the diameter for the cross sectional surface of a cylinder cut at different tilt angles. Note the non-linear relationship between ellipse tilt angle and progressive diameter overestimation, especially for tilt angles over 30 degrees. (**H**) Heat map demonstration of the non-linear error introduced by an increasing difference between the two perpendicular diameters of an ellipse, Diameters 1 and 2, when the formula for a circle is used to calculate the diameter. (**I**) Summary of proposed SAE approach to calculate the minor diameter of an ellipse. The long-established formula for ellipse area (Eq. (1)) is combined with a more recently developed infinite series formula for determine ellipse perimeter^56^ (Eq. (2)). The SAE approach is based on the notion that an elongated 2-dimensional surface with a known area and perimeter corresponds to a unique ellipse shape. The SAE equation is achieved by replacing R in Eq. (1) with A_e_/πr and expanding the first four terms of the infinite series (Eq. (3)). The equation is solved for r for a shape with known area and perimeter, and the minor diameter for the ellipse is next calculated as 2r. A_e_ = area of ellipse; R = long radius; r = short radius; p = perimeter of ellipse; h = (R − r)^2^/(R + r)^2^. (**J**) Violin plots of fiber diameter distributions. The violin plots show distributions myelinated fiber distributions with median and quartiles for diameters obtained by the SAE approach as well as for minimum Feret diameters and circle-based diameters after determining fiber area or perimeter. Note significant overestimations of fiber diameters when traditional methods were applied.

Left-sided L6-S3 VRs were removed intra-operatively and processed for light microscopy (LM) (Supplementary Fig. S1). Initial LM studies indicated marked size and shape variations for myelinated axons of the L6–S3 VRs (n = 6 subjects) (Fig. 1A–C). Quantitative assessments of myelinated fiber shape by multiple methods showed similar deviations from a circular shape for all VRs (Fig. 1D, Supplementary Fig. S2). Pooled data across the L6–S3 VRs computed the shape factor, form factor, aspect ratio, compactness, and roundness as 3.87 ± 0.02, 0.86 ± 0.01, 0.72 ± 0.01, 0.82 ± 0.01, and 0.69 ± 0.01 respectively. Corresponding values for a circle are 3.54 for the shape factor and 1 for the other outcome measures. Collectively, extensive fiber shape heterogeneity and inclusion of numerous non-circular fibers were demonstrated.

Our new tool to assess nerve fiber size is referred to as a shape-adjusted ellipse (SAE) approach to determine fiber diameter and is based on five notions: (1) Natural nerve fiber shape is regarded as a cylinder, i.e. a geometric figure with parallel sides and circular bases^12,13^; (2) transverse sectioning of a cylinder at an acute angle results in an ellipse-shaped cut surface (Fig. 1E); (3) sectioning of a cylinder at increasing tilt angles results in an ellipse with progressively increasing cut surface area in a non-linear relationship (Fig. 1F–H); (4) the minor axis length of an ellipse is identical to the diameter of the circular base, regardless of tilt angle or direction (Fig. 1F); (5) an ellipse with a known perimeter and surface area represents a unique geometrical shape, and its minor axis length can be calculated (Fig. 1I).

We next applied multiple methods to determine the diameter of myelinated fibers across L6–S3 VRs. Compared to the SAE approach, the minimum Feret, area circle, and perimeter circle approaches significantly overestimated fiber diameters by 15.1%, 32.1%, and 42.9%, respectively (Fig. 1J, Supplementary Table S1).

**Figure 2:**
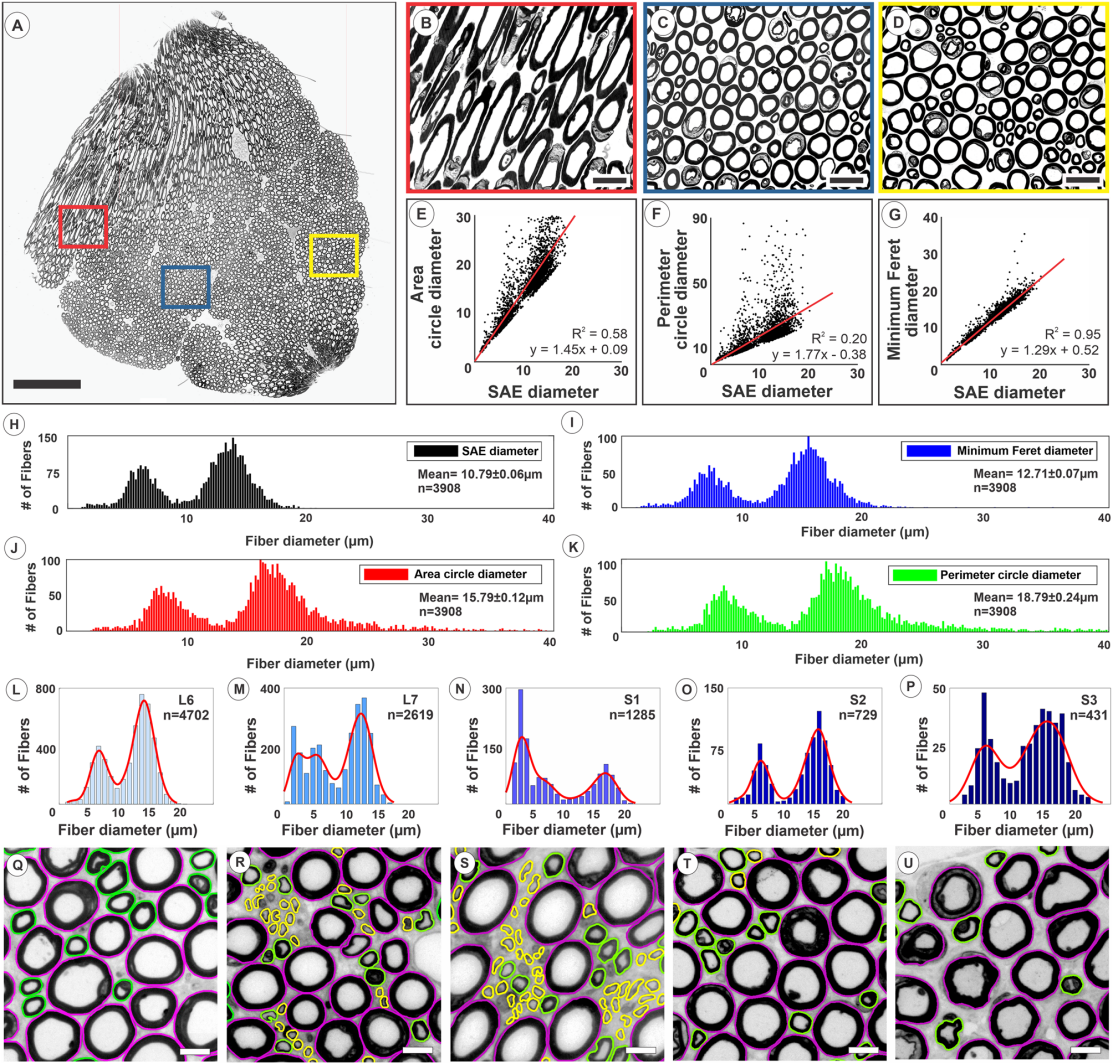
Validation of myelinated nerve fiber size determination using a SAE approach. (**A**) Light micrograph of a transverse section of an L6 VR in a rhesus macaque. Segmentation of all myelinated axons in the micrograph showed a total of 3,908 fibers. Red, blue, and yellow boxes show regions of interest depicted at higher magnification in (**B–D**) Note elongated fibers merging into main body of this proximal VR segment in (**B**). (**E–G**) Correlation graphs between calculated fiber diameter after SAE correction and corresponding diameters obtained by determining the Feret minimum diameter in or calculating the diameter based on the formula for the area or perimeter of a circle. Note dispersion of fiber diameters, especially for large diameter fibers based calculations using the circle formula approaches. (**H–K**) Frequency distribution graphs for myelinated fiber diameters determined using the SAE approach, Feret minimum diameter, and by calculating the diameter based on the formula for the area or perimeter of a circle. Note right-shift to larger diameters for both the γ- and α-motor fiber peaks and overestimation of diameters to non-physiologic range in estimates based on the formula for a circle. (**L–P**) Frequency distribution graphs for fiber diameters of myelinated axons in the L6–S3 VRs of one representative rhesus macaque. (**Q–U**) Representative areas of L6–S3 corresponding ROIs for segmented contours of myelinated fibers. Fibers with a diameter range of 0–4 µm are depicted in yellow, > 4–10 µm in green, and > 10 µm in magenta. Note presence of predominantly the smallest fibers of the 0–4 µm diameter range, corresponding to myelinated autonomic fiber size, in the L7 and S1 VRs in this subject. Scales: 500 µm in (**A**); 50 µm in (**B–D**); 20 µm in (**Q–U**).

**Figure 3:**
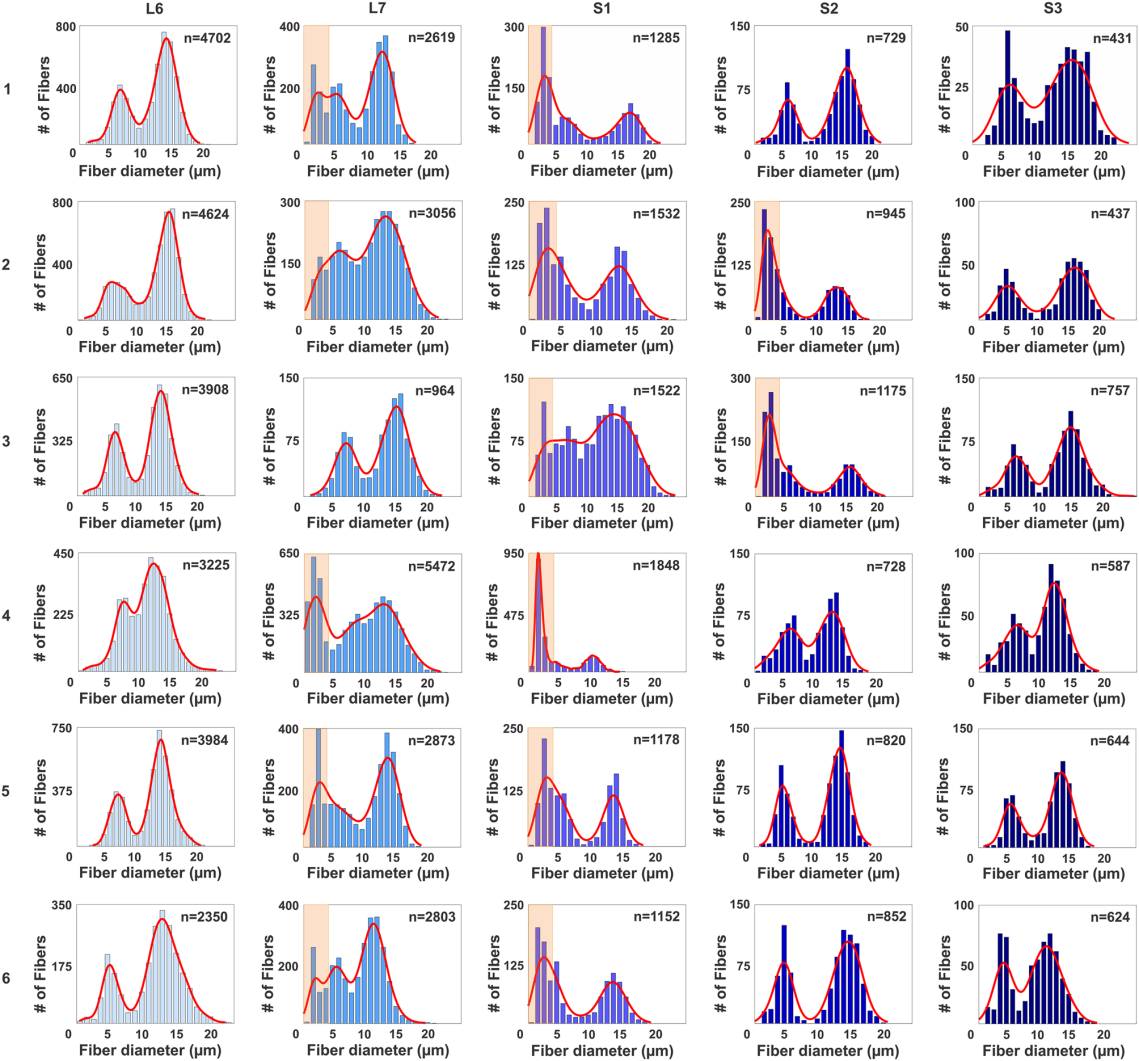
Myelinated fiber size and distribution across lumbosacral ventral roots (VRs) in rhesus macaques. Display of myelinated fiber size distribution for L6–S3 VRs in six female rhesus macaques. Note bimodal size distribution in all of the L6 and S3 VRs with size peaks corresponding to the large myelinated axons represented by γ- and α-motor fibers^21–23^. Within the L7–S2 VR span, all subjects show an additional peak in 2 or 3 consecutive roots of small-sized myelinated axons, highlighted by salmon-colored boxes for fibers within the 0–4 µm range. This population of fibers correspond to the small diameter of preganglionic parasympathetic fibers^24^. Note extensive individual variation for the rostro-caudal distribution of the autonomic fiber population with detection of this cohort of fibers predominantly in the L7 + S1 VRs (subjects 1, 4, 5, and 6), L7 + S1 + S2 VRs (subject 2), or S1 + S2 VRs (subject 3).

Regression analysis showed the best fit between the SAE diameter and diameters determined by the minimum Feret, area circle, and perimeter circle approaches, and numerous outliers were identified, especially for the circle-based approaches (Fig. 2A–G). Graphical representations of VR fiber size showed, compared to the SAE approach, a right-shift for the traditional methods, especially for circle-based methods. (Fig. 2H–K). The SAE approach was subsequently applied to determine the size distribution of myelinated axons across the L6–S3 VRs in individual subjects (n = 6) (Figs. 2L–U, 3). Graphical representation of the fiber size distribution showed a bimodal distribution of myelinated fibers in the α- and γ-motor fiber range in all L6 and S3 VRs. However, the rostro-caudal size distribution of fibers in the small diameter range of pre-ganglionic autonomic fibers varied extensively, as the additional fiber distribution peak was restricted to the L7 + S1 VRs (n = 4), S1 + S2 VRs (n = 1), or across L7 + S1 + S2 VRs (n = 1) (Fig. 3).

**Figure 4:**
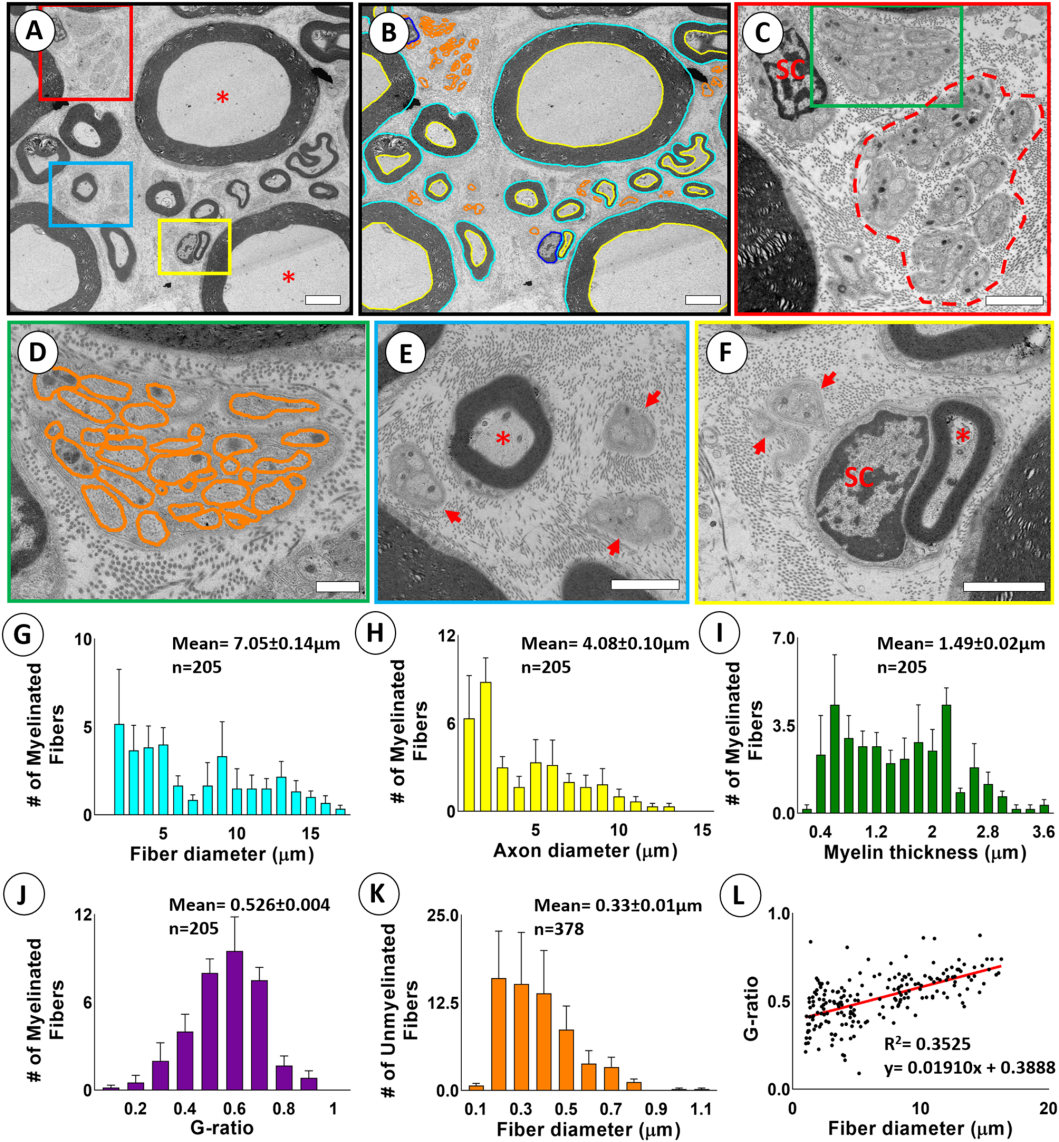
Determination of size and myelination of primate ventral roots using SAE correction in TEM. (**A**) Representative electron micrograph from an S2 VR demonstrating myelinated fibers of varying sizes and myelination and unmyelinated axons. *Indicates large myelinated axon. (**B**) Segmented electron micrograph with outer and inner contours of myelinated fibers in blue and yellow, respectively. Contours of unmyelinated fibers are in orange. (**C**) Detail of boxed are in (**A**) showing cluster of unmyelinated fibers. (**D**) Remak bundles of unmyelinated fibers in green box in (**C**) with surrounding collagen fibers. (**E**) Detail of blue box in A. with small myelinated fiber and adjacent unmyelinated fibers. (**F**) Detail of small myelinated fiber indicated by * and Schwann cell nucleus (SC) in yellow box in (**A**). (**G–K**) Frequency distribution graphs of fiber diameter, axon diameter, myelin thickness, and G-ratio for 205 myelinated fibers and fiber diameter for 378 unmyelinated fibers from representative region of interests (ROIs) in S2 VRs of rhesus macaques (n = 6). (**L**) Graph depicting correlation between fiber diameter and G-ratio of 205 myelinated fibers pooled from representative ROIs in S2 VRs of rhesus macaques (n = 6). Scales: 4 µm in (**A,B**); 2 µm in (**C**); 600 nm in (**D**); 2 µm in (**E**); 2 µm in (**F**).

Tissue blocks for the S2 VRs (n = 6) were processed for ultrastructural studies, and transmission electron microscopy (TEM) determined the shape and size of unmyelinated axons (Supplementary Fig. S3). Electron micrographs showed numerous unmyelinated axons of varied shape, interspersed between larger myelinated axons and collagen fibers (Fig. 4A–F). The shape factor, form factor, aspect ratio, compactness, and roundness were computed for the unmyelinated axons as 4.02 ± 0.06, 0.80 ± 0.02, 0.59 ± 0.02, 0.74 ± 0.02, and 0.56 ± 0.03 (n = 6), respectively, and they were markedly different from corresponding values for a circle (Supplementary Fig. S4A–F). The findings show that unmyelinated fibers, similar to myelinated fibers, exhibit extensive shape heterogeneity with the inclusion of many non-circular axons.

Next, we used the SAE approach to determine the diameter for S2 VR myelinated and unmyelinated axons in a representative region of interest (n = 6). For determining the G-ratio, a code was developed to link the centroid locations for the outer and inner myelin contours of individual segmented fibers. A broad distribution range was suggested for the axon and fiber diameters and myelin thickness, whereas a single peak pattern was showed for the G-ratio (Fig. 4G–J). The unmyelinated axons showed markedly smaller axon diameters than those of the smallest myelinated fibers (Fig. 4K). For myelinated fibers, the G-ratio was positively correlated to fiber diameter (Fig. 4L).

The SAE approach was also applied to show feasibility for determining nerve fiber size and myelination within nervous tissue undergoing injury-induced degenerative changes. For this purpose, we examined the avulsed distal segment of S2 VRs at 2, 12, and 18 months after an L6–S3 ventral root avulsion (VRA) injury (Supplementary Fig. S5). Following a VRA-induced Wallerian degeneration of efferent motor and autonomic axons within the avulsed S2 VR segment, quantitative studies showed a gradual post-operative re-population of the avulsed S2 VR by myelinated fibers. A parallel decrease over time in the number of myelinated fibers with degenerative features was also noted in the avulsed S2 VR segments.

## Discussion

The present study introduced the SAE approach as a new tool to determine nerve fiber diameter, regardless of fiber dispersion angle, and has shown utility for the size analysis of both myelinated and unmyelinated fibers. Traditional methods commonly apply the formula for a circle to calculate the diameter of a nerve fiber after initial measurement of the fiber cross-sectional area^8,10,11,14,15^ or the fiber perimeter^9,12^. The convex shape of an axon may also be taken into consideration when determining axonal size. For instance, the smallest distance between a pair of antipodal points belonging to the convex hull of the axon has been used to estimate nerve fiber diameters^25^, and the minimum Feret diameter has been used as a caliper measurement of nerve fiber size^26^. However, these earlier methods do not consider the dispersion angle formed between each individual fiber segment’s direction and the mean direction of the overall nerve bundle. Instead, the present SAE approach, which corrects for the fiber dispersion angle, markedly reduces the number of outliers and data scatter, especially when compared to methods using the cross-sectional perimeter or area measurements and the formula for a circle to determine fiber size.

The functional organization of the lumbosacral spinal cord in large mammals shows extensive variability between subjects. Early mapping of lumbosacral VRs in rhesus macaques showed that specific motor fiber innervation of lumbosacral roots varies en bloc along the rostro-caudal length of the spinal cord, resulting in a pre- or post-fixed organizational phenotypes^27^. Similar shifts along the rostro-caudal distribution of the spinal cord has been shown for the location of specific motor nuclei, which maintain the relative distances to adjacent motor nuclei^28,29^. Our findings expand the notion of rostro-caudal variability of efferent projections from the spinal cord between subjects, and that it also applies to preganglionic parasympathetic fibers of the autonomic nervous system in primates.

Neuromodulation strategies, including lumbosacral nerve root and spinal cord electrical stimulation, have emerged and shown promise in attempts to map or augment lower urinary tract function in animal models and humans. Such strategies include sacral nerve stimulation^30,31^, sacral anterior root stimulation (SARS)^32,33^, epidural stimulation^34,35^, and transcutaneous spinal cord stimulation^36,37^. In clinical practice, neuromodulation has demonstrated promise for the treatment of an overactive bladder syndrome but symptom relief and overall treatment success vary between patients^38^. Improved anatomical mapping tools, including the SAE approach for fiber size corrections, to determine normal variability of autonomic nerve fiber distributions in lumbosacral roots may facilitate development of refined neuromodulation strategies for relieving neuro-urological conditions.

Studies of nerve regeneration to overcome paralysis in experimental models include morphological examination of myelinated and unmyelinated axons, which may extend across denervated tissues in the spinal cord or peripheral nervous system. Signs of regeneration in the nervous system include injury-induced branching of fibers and axonal extensions, which are under the influence of local guidance molecules and characteristically show a multitude of growth patterns and directions^39–41^. Regenerating axons may therefore show an especially large variety of dispersion angles at cut surfaces of intramedullary fiber tracts and peripheral nerves sectioned in the transverse plane. Here, we show examples of avulsed S2 VRs, which have undergone Wallerian degeneration followed by re-population of the distal VR segment by myelinated axons. Although the proximal end of the avulsed VR segment remained separated from the spinal cord, the VRs were gradually repopulated by myelinated axons over time. These findings are consistent with prior reports suggesting innervation of denervated VRs by sensory fibers, possibly including U-turning ventral root afferents^42,43^. The SAE approach to determine fiber size and myelination across injury and disease models will allow for improved predictions of functional properties in translational studies of neural repair and nerve regeneration.

We conclude that our SAE approach for size correction of cylindrical structures in sectional planes provides a useful and novel tool determining size and myelination of segmented nerve fibers in light and electron microscopy. The SAE approach allows for non-biased data inclusion and size correction of all myelinated and unmyelinated nerve fibers, regardless of fiber dispersion angle. SAE corrections of fiber size and myelination provides refined ground truth data on the morphological signatures of subsets of fibers in mixed nerves and white matter tracts. SAE-corrected fiber size measurements may also contribute to refined nerve stimulation models for emerging strategies to reverse conditions by neuromodulation in translational and clinical research studies. In addition, the SAE approach may have expanded utility for size corrections of all tubular structures imaged in oblique planes in biological tissues, including blood vessels, excretory ducts, and airways, not only by microscopy, but also when using non-invasive imaging strategies.

## Methods

The present study was performed using archived lumbosacral ventral root tissues from female rhesus macaques *(Macaca mulatta)*. The subjects were 9.0 ± 0.7 years old and weighed 8.09 ± 0.79 kg (n = 6). The nerve root samples were originally harvested as control samples during a ventral root injury procedure in connection with separate research studies on cauda equina and conus medullaris forms of spinal cord injury in rhesus macaques^44,45^. A brief description of the original tissue harvesting is provided below.

### Animal procedures

All animal procedures were performed at the California National Primate Research Center (CNPRC), University of California at Davis. The institution is accredit by the Association for Assessment and Accreditation of Laboratory Animal Care (AAALAC) and all procedures were approved by the UC Davis Institutional Animal Care and Use Committee (IACUC). In addition to the certification already cited all animal procedures and care were performed in compliance with the Guide for the Care and Use of Laboratory Animals provided by the Institute for Laboratory Animal Research^46^. Guidelines for ARRIVE 2.0 for the care and use of laboratory animals were also followed^47–49^.

Lumbosacral ventral roots (VRs) were harvested after a pre-operative lumbar magnetic resonance imaging (MRI), spine radiographs, and intra-operative identification of individual segmental levels. Pre-operative imaging was performed to determine the relationship between the vertebral column and spinal cord and to plan spine surgery. A surgical plane of anesthesia was established by 1–2% isoflurane in O_2_ via endotracheal tube and 7–10 µg/kg/min fentanyl iv. Following a skin incision over the L1–L5 spinous processes, a left-sided laminectomy of the caudal aspect of the L1 vertebra to the rostral part of the L3 vertebra, and opening of the dura, the L6-S3 VRs were identified based on anatomical landmarks and size characteristics^43–45,50^. The left L6–S3 VRs were avulsed from the surface of the spinal cord by gentle traction using a pair of fine forceps, and an approximately 5 mm segment of each avulsed L6–S3 VR was removed and placed in a solution containing 2% paraformaldehyde (PF) and 2.5% glutaraldehyde (Glut) in phosphate buffered saline (PBS, pH 7.4), for tissue fixation and subsequent processing for light and electron microscopy. The distal portion of each L6–S3 VR was deflected from the spinal cord, the dura closed using a continuous 6-0 ETHILON suture, the paraspinous muscles and fascia closed in layers, and the skin closed using 4-0 VICRYL sutures. All animals recovered post-operativelyn.

In three subjects, the spinal cord and associated lumbosacral nerve roots were harvested post-operatively at 2 months (n = 1), 12 months (n = 1), and 18 months (n = 1) to provide VRs for the present studies. The post-op tissues were collected after a necropsy procedure, which included intravascular perfusion using a 4% PF solution. The lumbosacral VRs were dissected out and post-fixed for 24 h in a 2% PF + 2.5% *Glut solution in PBS.*

### Light microscopy and shape analysis

Following the intra- and post-operative harvesting of the L6-S3 VR segments, the tissues were prepared for light microscopic analysis according to our proposed pipeline for tissue processing, imaging, and analysis (Supplementary Fig. S1). In short, after the immersion fixation in a 2% PF + 2.5% Glut solution in PBS, the VR segments were rinsed in phosphate buffer, osmicated in 1% osmium tetroxide (OsO4), rinsed in aqueous solution, dehydrated in ascending concentrations of ethanol, infiltrated in 50% propylene oxide (PO) + 50% Epon plastic resin, and embedded in 100% Epon (Supplementary Fig. S1, steps 1–5). Next, semi-thin transverse sections were cut at 0.5 µm thickness and stained with a 1% toluidine blue solution (Supplementary Fig. S1, step 6). All tissue sections were inspected by light microscopy before inclusion into the analysis to ensure visualization of the full circumference of the epineurium and anatomical integrity of all fascicles within each VR. Serial light microscopic images of the entire VR cross section were obtained at × 100 magnification, and the images were tiled to generate a photo-montage using a Nikon E600 light microscope equipped with a DS-Fi3 camera and Nikon NIS-Elements software (Supplementary Fig. S1, steps 7–8). The contour of each myelinated fiber for the L6–S3 VRs in all subject was segmented with manual or supervised approaches using Fiji ImageJ^51^, or Neurolucida 360 (MBF Bioscience) to determine the cross-sectional area, perimeter as well as the maximum and minimum Feret diameters for subsequent shape analysis of all myelinated fibers (Supplementary Fig. S1, step 9). Next, an array of five established metrics were used to perform mathematical descriptions of shape, previously developed and validated across multiple disciplines, including pathology, paleontology, and social science to determine the shape of e.g. tumors, bones, calcifications, and geographical objects^52–59^. We determined shape factor, form factor, aspect ratio, compactness, and roundness to assess how closely each myelinated fiber may correspond to the shape of a circle according to the formulas and descriptions below (Supplementary Fig. S1, step 10).

**Table:** 

Shape features	Formula	Description
Shape factor	$\frac{Perimeter}{\sqrt{Area}}$	Ratio between the perimeter of the shape and the square root of the area. For a circle, the shape factor is equal to 3.54
Form factor	$\frac{4\pi Area}{{Perimeter}^{2}}$	Ratio between the area of the shape and the area of a circular convex hull calculate from the perimeter. For a circular shape, the form factor is equal to 1
Aspect ratio	$\frac{MinFeret}{MaxFeret}$	Ratio of the minimum Feret diameter to the maximum Feret diameter of the shape. For a circle, the aspect ratio is equal to 1
Compactness	$\frac{\sqrt{\frac{4}{\pi }Area}}{MaxFeret}$	Ratio between the radius of a circle based on the shape Area divided by the maximum Feret diameter. Compactness of a circle is equal to 1
Roundness	$\frac{4Area}{\pi {MaxFeret}^{2}}$	Ratio between the area of the shape and the area of a convex hull encompassing the shape. A perfect circle has roundness value equal to 1

### Morphometric analysis

Light microscopic analysis were performed to determine shape and size of myelinated fibers. To accommodate for myelinated axonal profiles, which deviate from a circular shape in the sectional plane, a new approach to determine fiber size and myelination was developed. Here, all myelinated fibers are considered to have the shape of a cylinder with a circular base, and the cross-sectional profile of each fiber was considered to present the area and perimeter of an ellipse with a dispersion angle between the direction of the individual fiber and the overall orientation of the VR segment. The dispersion, or tilt, angle can be estimated.

For the ellipsoid shape, the semi-minor axis r is equal to the radius of the cylinder’s base, thus the semi-major axis R can be defined using r by $\text{R}=\text{r}/{\cos}\left(\uptheta \right)$. Consider the formula for ellipse area $\text{Ae}={\uppi \times r \times R}$, where r is the length of the semi-minor axis and R is the length of the semi-major axis of the ellipse. Both R and r originate at the center of the ellipse. Replacing R by $\text{r}/\cos\left(\uptheta \right)$, the ellipse area can be rewritten as $\text{Ae}={\uppi \times r \times r}/{\cos}\left(\uptheta \right)$. In this way, the ellipse area is related to the tilt angle (θ) and relation between the ellipse area ($\text{Ae}$) and the area of the original circle-shape $\text{Ac}=\uppi {\text{r}}^{2}$ can be expressed as $\text{Ae}=\rho\text{ Ac}$ , where $\uprho = 1/{\cos}\left(\uptheta \right)$. The parameter ρ computes the error introduced when applying the circle approach to calculate the size of a nerve fiber. Note that, for the cases where the nerve fiber is a perfect circle, ρ value is equal to one and there is no difference between the approaches.

It is possible to calculate the smallest diameter of ellipse combining the formula for the area of an ellipse (1)

1 $$ R = \frac{{{\text{Ae}}}}{\pi x r} $$

and an infinite series formula for the ellipse perimeter (2)^60^

2 $$ {\uprho } = {\uppi }\left( {{\text{r}} + {\text{R}}} \right)\mathop \sum \limits_{{{\text{n}} = 0}}^{\infty } \left( {\begin{array}{*{20}c} {0.5} \\ n \\ \end{array} } \right)^{2} {\text{h}}^{{\text{n}}} $$

where $\text{h}=\frac{{\left(\text{R}-\text{r}\right)}^{2}}{{\left(\text{R}+\text{r}\right)}^{2}}$. The Eq. (3) is achieve by replacing R in (2) by (1) and expanding the first four terms of the infinite series

3 $$ {\uprho } = {\uppi }\left( {\frac{{{\text{Ae}}}}{{{\pi r}}} + {\text{r}}} \right)\left( {1 + \frac{1}{4}{\text{h}} + \frac{1}{64}{\text{h}}^{2} + \frac{1}{256}{\text{h}}^{3} } \right) $$

where $$\text{h}=\frac{\uppi {\text{r}}^{2}-\text{Ae}}{\uppi {\text{r}}^{2}+\text{Ae}}$$.

When the values for the area and perimeter are known, the length of the semi-minor axis *r* is calculated by solving Eq. (3) using the Newton method. The value of the minor axis, i.e. fiber diameter, is next calculated as two times the value of r.

We have developed a code to calculate *r* and the smallest fiber diameter using our shape-adjusted ellipse (SAE) approach in MATLAB. All MATLAB codes used in the calculations, the input and output data, information regarding their development, and directions for code use are available at www.github.com/petrabartmeyer/ShapeAdjustedEllipse.

For SAE approach validation and comparisons across methods, the size of myelinated axons was also determined by implementing long-established approaches of calculating the fiber diameter using the formula for a circle and measurements of the cross-sectional area^8,10,11,14,15^ or perimeter^9,12^. Additional comparisons were made with fiber size estimates based on the previously established approach of determining the minimum Feret diameter for each myelinated axon^26^. Linear regression analysis was performed to determine the coefficient of determination, R^2^, with fiber size based on our SAE approach as the dependent variable and fiber size obtained by a traditional method as the independent variable.

### Electron microscopy

Ultrathin sections (70–90 nm), from the segments of the S2 ventral nerve root previously embedded in resin (n = 6) were obtained in an ultramicrotome (RMC PowerTome Ultramicrotome, Boeckeler Instruments), collected on formvar-coated single slot grids, contrasted with uranyl acetate and lead citrate (Supplementary Fig. S3, steps 1–8). The sections were analyzed under a transmission electron microscope operating at 80 kV (Tecnai G2 Spirit Twin, FEI, ThermoFisher Scientific, or Hitachi H-7500). A region of interest (ROI) composed by 52 electron micrographs at 1500X magnification (average area = 6135.67 ± 89.87 µm^2^) from the S2 VRs of each animal (n = 6) was photographed using a Gatan Orius SC 1000B digital camera (Gatan, Inc.) or AMT camera (Supplementary Fig. S3, step 9). The tiling of the ROI images were performed by Adobe Photoshop (version: 21.1.3 20200508) or Image Composite Editor (ICE, Microsoft) (Supplementary Fig. S3, step 10). Data segmentation of each myelinated and unmyelinated axon was performed with manual or supervised approaches using Fiji ImageJ^51^, or Neurolucida 360 (MBF Bioscience). Both programs generated raw data files for the area, perimeter, centroid, as well as the maximum and minimum Feret diameters for each nerve fiber (Supplementary Fig. S3, step 11). The shape factor, form factor, aspect ratio, compactness, and roundness of all unmyelinated fibers were next determined within the ROI for each S2 VR (Supplementary Fig. S3, step 12).

The outer and inner contours of the myelin for each myelinated fiber were also segmented to allow for the calculation of both the fiber and axon diameters. Calculations of myelin thickness and the G-ratio, as the ratio between the diameter of the axon proper and diameter of the corresponding myelinated fiber, require linkage between each axon and fiber pair. Although both Fiji ImageJ^51^ and Neurolucida 360 allow for the differentiation between segmented fiber and axon structures, neither one of the software products can automatically link an axon structure to its corresponding fiber structure. To overcome this limitation and to allow for the calculation of myelination parameters, we developed an assignment routine based on the centroids of the structures. The routine calculates the Euclidean distance between each axon structure and all the fiber structures. This procedure creates the distance matrix used to guide the assignment problem. The solution of the assignment problem returns the optimal match between axon and fiber structures. The assignment routine was implemented in MATLAB using the Hungarian method to solve the assignment problem. The routine is available at Github repository at www.github.com/petrabartmeyer/ShapeAdjustedEllipse.

The segmented data was used for light microscopic and ultrastructural analysis, including SAE morphometric calculations of fiber diameter, axon diameter, myelin thickness (myelin thickness = (fiber diameter − axon diameter)/2)), and G-ratio (G-ratio = axon diameter/fiber diameter) for myelinated axons (Supplementary Fig. S1, steps 11, 12; Supplementary Fig. S3, steps 13, 14).

### Statistics

All data are presented as mean ± standard error (SE). Methods to determine fiber diameter were compared using repeated measures one-way ANOVA with the Geisser–Greenhouse correction. This was followed by Tukey’s multiple comparisons test with individual variances computed for each comparison. Linear regression analysis was performed to determine the coefficient of determination, R^2^, as a goodness-of-fit measure between dependent and independent variables. The non-parametric ANOVA One-way testing followed by the Kruskal–Wallis test and Dunn’s multiple comparison test were performed for comparisons between groups using GraphPad Prism 8, version 8.4.3 (GraphPad Software, Inc, La Jolla, CA) https://www.graphpad.com/. A value of p < 0.05 was considered to reflect a statistically significant difference between groups. Frequency distributions for all fiber shape and morphometric size analyses were calculated and plotted using GraphPad Prism 8, version 8.4.3, https://www.graphpad.com/. An interpolation curve frequency distribution analysis was also included for the frequency distribution of fiber size in individual VRs, and the analysis was coded in MATLAB, using the function “histfit”. The distribution function considered the option “kernel”, which uses a nonparametric kernel-smoothing distribution strategy.

## Supplementary information

Supplementary information.

## Data Availability

Any raw data supporting the current study is available from the corresponding author upon reasonable request.

## Code availability

The code used in the calculations, the input and output data, information regarding their development, and directions for code use are available at www.github.com/petrabartmeyer/ShapeAdjustedEllipse.
