# Supplementary material for: A shape-adjusted ellipse approach corrects for varied axonal dispersion angles and myelination in primate nerve roots
Source: Sci Rep. 2021 Feb 4;11:3150. doi: 10.1038/s41598-021-82575-9 (PMC7862494; doi:10.1038/s41598-021-82575-9)
Supplement: Supplementary file 1 — Supplementary information. [file 41598_2021_82575_MOESM1_ESM.pdf]

Supplementary Information

A Shape-Adjusted Ellipse Approach Corrects for Varied Axonal Dispersion Angles and Myelination in Primate Nerve Roots

Petra M. Bartmeyer[1,2], Natalia P Biscola[1,3], Leif A. Havton[4,5,6]*

[1] Department of Neurology, David Geffen School of Medicine at UCLA, Los Angeles, CA, USA

[2] School of Electrical and Computer Engineering at University of Campinas, Campinas, SP, Brazil

[3] Department of Neurology, Icahn School of Medicine at Mount Sinai, New York, NY, USA

[4] Departments of Neurology and Neurobiology, David Geffen School of Medicine at UCLA, Los Angeles, CA, USA

[5] Departments of Neurology and Neuroscience, Icahn School of Medicine at Mount Sinai, New York, NY, USA

[6] Neurology Service and RR&D National Center for the Medical Consequences of Spinal Cord Injury, James J. Peters Veterans Administration Medical Center, Bronx, NY, USA

*Corresponding Author:

Leif A. Havton, M.D., Ph.D.
Department of Neurology
Icahn School of Medicine at Mount Sinai
New York, NY 10029
Email: leif.havton@mssm.edu

Supplementary Table S1. Summary of L6-S3 VR diameter measurements using multiple methods. A. Diameters of L6-S3 VRs were obtained by the SAE approach and compared to minimum Feret diameters and circle-based diameters after fiber area or perimeter measurements. **B.** Summary of Tukey's multiple comparisons test. Note significant overestimations of diameters when determining minimum Feret diameters or when using the formula for a circle after cross-sectional area or perimeter measurements.

A

Nerve root	SAE (µm)	Min Feret (µm)	Area circle (µm)	Perim circle (µm)
L6 (n=6)	11.42±0.24	13.35±0.18	15.23±0.38	16.64±0.71
L7 (n=6)	9.43±0.59	11.25±0.68	12.93±0.96	14.15±1.15
S1 (n=6)	7.64±0.87	8.64±1.01	10.29±1.45	11.33±1.85
S2 (n=6)	9.41±0.92	10.52±1.00	11.79±1.02	12.40±1.00
S3 (n=6)	10.78±0.54	12.28±0.45	14.08±0.57	15.08±0.58
Mean ±SE	9.74±0.65	11.21±0.80	12.87±0.86	13.92±0.94

B

Tukey's multiple comparisons test	Mean Diff.	95.00% CI of diff.	Summary	Adjusted P Value
SAE vs. Min Feret	-1.474	-2.223 to -0.7259	**	0.0046
SAE vs. Area circle	-3.13	-4.213 to -2.047	**	0.001
SAE vs. Perim circle	-4.185	-5.770 to -2.599	**	0.0015
Min Feret vs. Area circle	-1.656	-2.084 to -1.228	***	0.0003
Min Feret vs. Perim circle	-2.71	-3.653 to -1.768	**	0.0011
Area circle vs. Perim circle	-1.055	-1.598 to -0.5112	**	0.0048

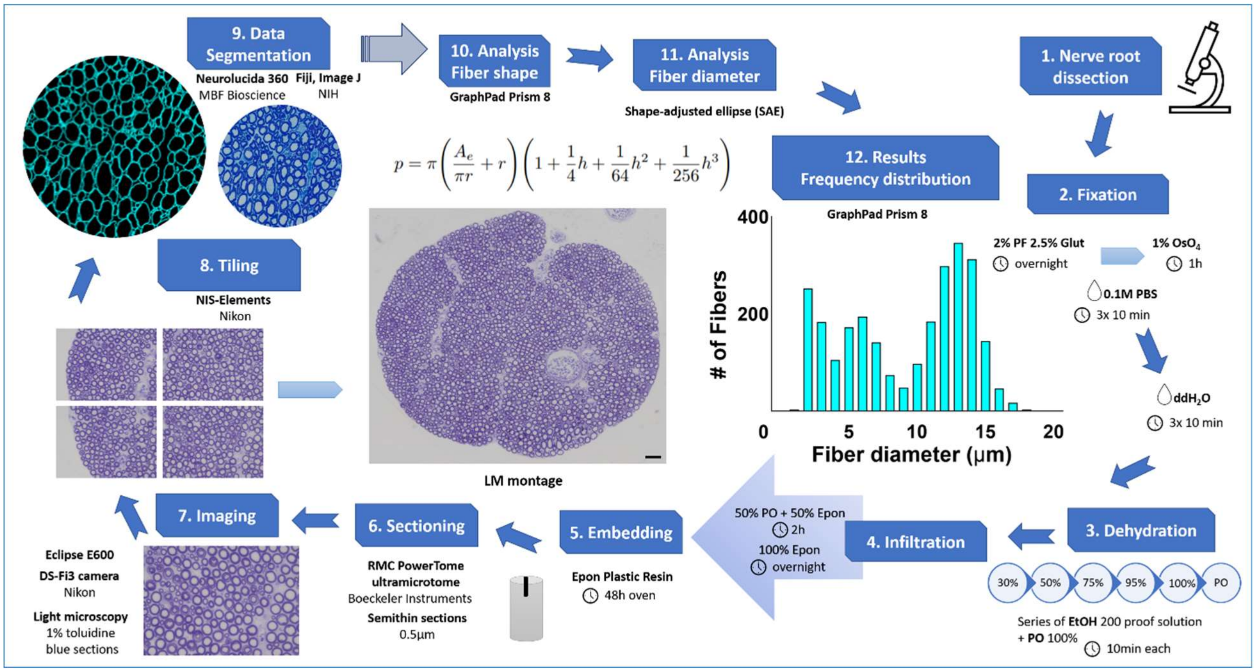

Supplementary Fig. S1. Pipeline for tissue processing and analysis of primate nerve roots to determine size of myelinated fibers using SAE correction in light microscopy (LM). Following intra-operative harvesting of lumbosacral ventral root (VR) segments [39], tissues are fixed in a 2% paraformaldehyde + 2.5% glutaraldehyde solution, rinsed in phosphate buffer, osmicated in 1% osmium tetroxide, rinsed in aqueous solution, dehydrated in ascending concentrations of ethanol, infiltrated in 50% PO + 50% Epon, and embedded in 100% Epon plastic resin. Next, semi-thin sections are cut at 0.5 μm thickness in the transverse plane and stained a with 1% toluidine blue solution. Serial light microscopic images of the entire VR cross section are captured at 100X magnification and images tiled to generate a photo-montage using a Nikon E600 light microscope equipped with a DS-Fi3 camera (Nikon). Montage shows a representative S2 VR. The contour of each myelinated fiber was segmented using ImageJ (NIH) [47] or Neurolucida 360 (MBF). The diameter for each fiber was calculated using the shape adjusted ellipse (SAE) approach to correct for the dispersion angle exhibited by fibers sectioned at an oblique plane. A quantitative display of myelinated fiber diameter is next displayed as a frequency distribution plot of a total of 852 fibers of representative S2 VR using a GraphPad Prism 8, version 8.4.3, https://www.graphpad.com/. Note two fiber size distribution peaks corresponding to γ- and α-motor fibers [21-23]. Scale bar in photo montage of S2 VR is 100 μm.

A

Nerve root	Shape factor	Form Factor	Aspect ratio	Compactness	Roundness
**L6** (n=6)	3.83±0.06	0.87±0.02	0.75±0.03	0.84±0.02	0.72±0.03
**L7** (n=6)	3.90±0.03	0.84±0.01	0.72±0.03	0.82±0.02	0.68±0.03
**S1** (n=6)	3.94±0.07	0.83±0.02	0.67±0.03	0.79±0.02	0.64±0.03
**S2** (n=6)	3.86±0.12	0.89±0.02	0.74±0.02	0.84±0.02	0.72±0.03
**S3** (n=6)	3.81±0.03	0.87±0.01	0.72±0.01	0.83±0.01	0.69±0.01
**Circle**	3.54	1.00	1.00	1.00	1.00

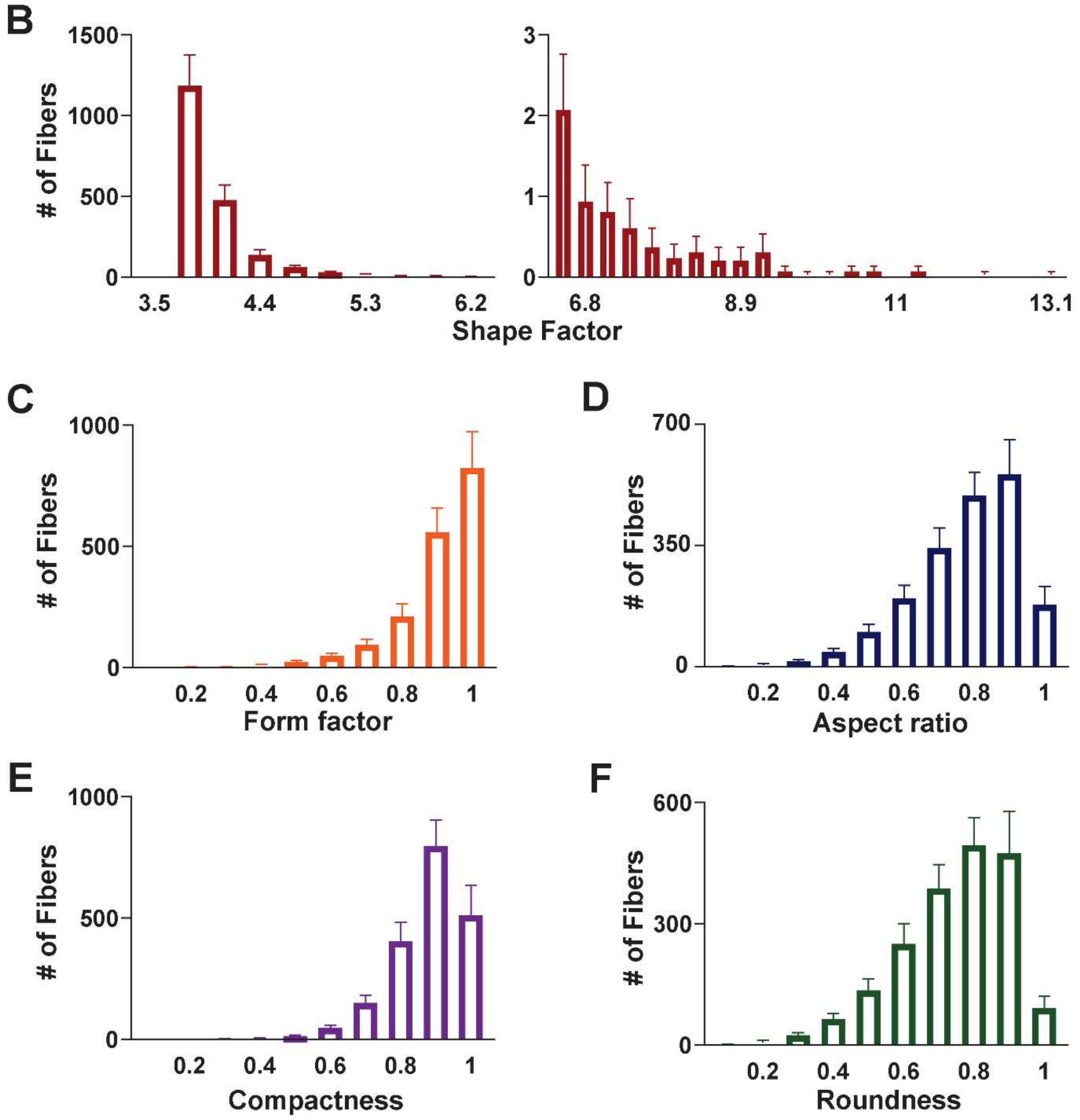

Supplementary Fig. S2. Multi-modal determination of myelinated nerve fiber shape in L6-S3 VRs. A. Table display of myelinated fiber shape determined for each segmental L6-S3 VR (n=6 subjects) using multiple shape outcome measures. Compared to the expected values for a circular shape, quantitative analysis of VRs for each segmental level suggested inclusion of ventral root fibers exhibiting non-circular shapes as determined by shape factor, form factor, aspect ratio, compactness, and roundness analysis. For each shape outcome measure, there was no statistical difference between the individual L6-S3 segmental levels by non-parametric ANOVA One-Way testing followed by Kruskal-Wallis test and Dunn's multiple comparison test. **B-F.** Frequency distribution data were pooled across the L6-S3 VRs in all subjects (n=6) for a total of 30 VRs and displayed as histograms for shape factor, form factor, aspect ratio, compactness, and roundness. Note consistent representation of a marked number of fibers exhibiting a non-circular shape regardless of shape outcome measure used for analysis.

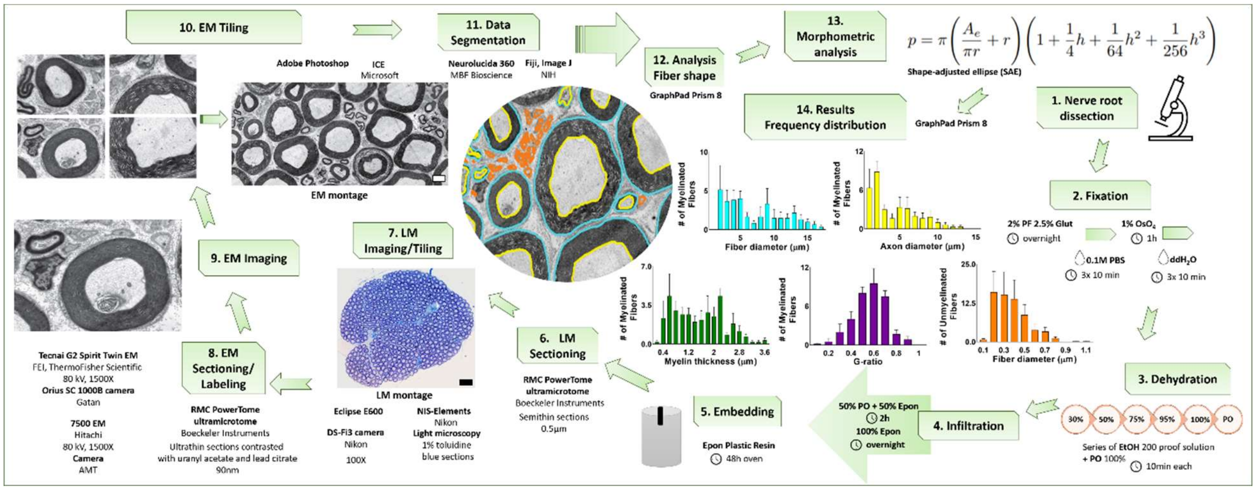

Supplementary Fig. S3. Pipeline for tissue processing and analysis of primate nerve roots to determine size of myelinated and unmyelinated fibers using SAE correction in transmission electron microscopy (TEM).

Following intra-operative harvesting of lumbosacral ventral root (VR) segments [39,45], tissues are fixed in a 2% paraformaldehyde + 2.5% glutaraldehyde solution, rinsed in phosphate buffer, osmicated in 1% osmium tetroxide, rinsed in aqueous solution, dehydrated in ascending concentrations of ethanol, infiltrated in 50% PO + 50% Epon, and embedded in 100% Epon plastic resin. Next, semi-thin sections are cut at 0.5 µm thickness in the transverse plane and stained a with 1% toluidine blue solution. Serial LM images of the entire cross section for a representative S2 VR are captured at 100X magnification, and images are tiled to generate a photo-montage using a Nikon E600 light microscope equipped with a DS-Fi3 camera (Nikon). The LM montage allows for an early evaluation of the technical quality of tissue morphology and section completeness, and identification of regions of interest. Ultrathin sections are cut at 60-90 nm thickness using an ultramicrotome equipped with a Diatome diamond knife, and sections are collected on formvar-coated one-hole copper grids. The collected sections are air dried and contrasted with uranyl acetate and lead citrate. Serial electron micrographs are collected at 1,500X magnification using a Tecnai G2 Spirit BioTwin TEM (FEI/Thermo) equipped with a Gatan Orius SC 1000B camera or a Hitachi 7500 TEM equipped with an AMT camera. The

digital images are tiled using Adobe Photoshop or Microsoft ICE software products. The outer and inner contour of the myelin sheath for each myelinated fiber and the contour unmyelinated axons are segmented separately using ImageJ (NIH) [47] or Neurolucida360 (MBF). The diameter for the outer and inner contours of myelinated fibers and the contour of unmyelinated fibers are calculated using the SAE approach to correct for the dispersion angle exhibited by fibers sectioned at an oblique plane. Results are presented as frequency distribution graphs for the fiber and axon diameters, myelin thickness, and G-ratio for myelinated fibers and as fiber diameter for unmyelinated fibers using a GraphPad Prism 8, version 8.4.3, https://www.graphpad.com/. Scale bar indicates 50 μm in the light micrograph montage and 10 μm for the electron micrograph montage.

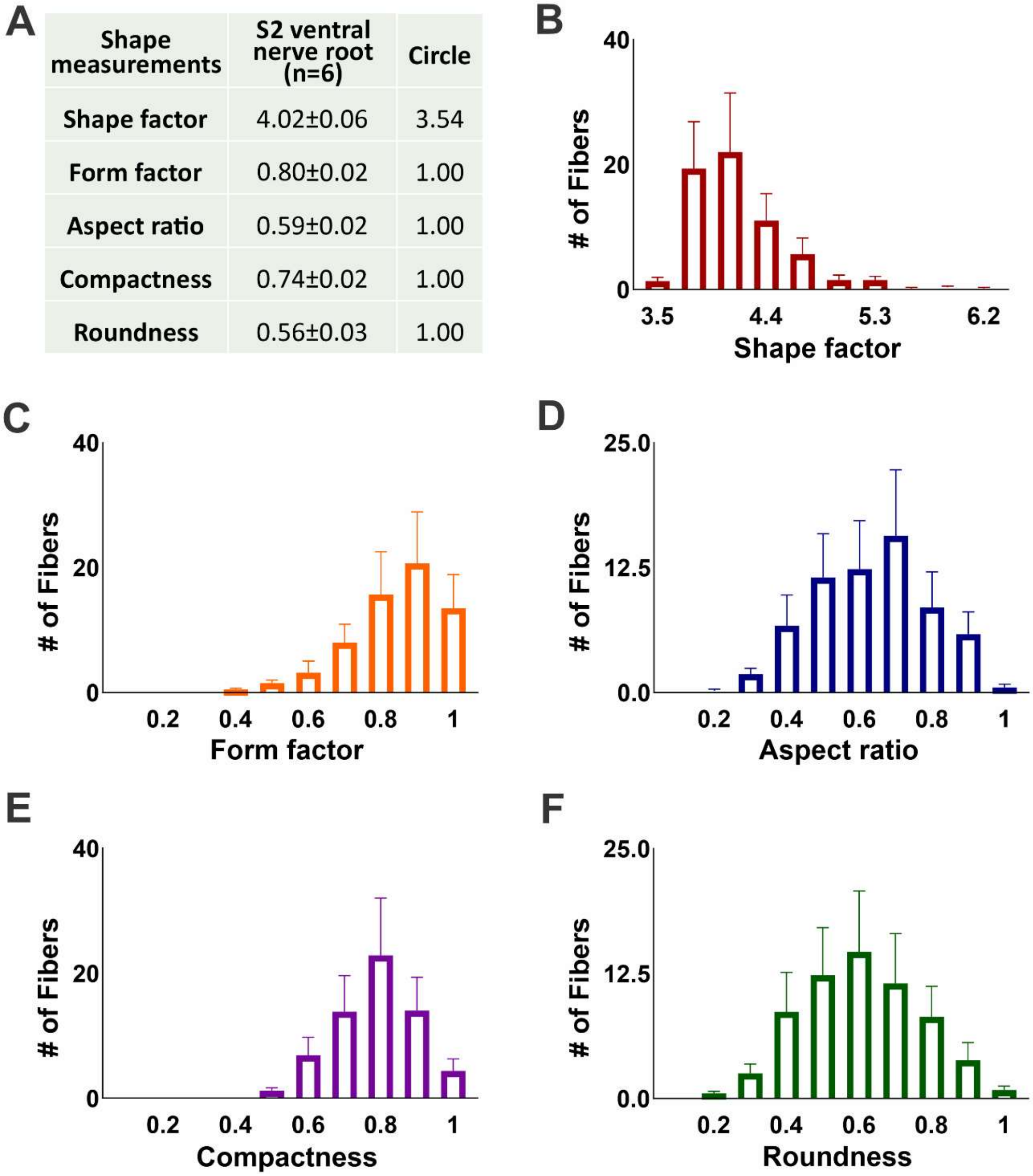

Shape measurements	S2 ventral nerve root (n=6)	Circle
Shape factor	4.02±0.06	3.54
Form factor	0.80±0.02	1.00
Aspect ratio	0.59±0.02	1.00
Compactness	0.74±0.02	1.00
Roundness	0.56±0.03	1.00

Supplementary Fig. S4. Determination of unmyelinated fiber shape in primate S2 VRs by multiple methods. A. Table display of unmyelinated fiber shape determined for S2 VRs (n=6 subjects) using multiple shape outcome measures. Compared to the expected values for a circular shape, quantitative analysis of S2 VRs for each segmental level suggested inclusion of ventral root fibers exhibiting non-circular shapes as determined by shape factor, form factor, aspect ratio, compactness, and roundness analysis. **B-F.** Frequency distribution histograms for shape factor, form factor, aspect ratio, compactness, and roundness of S2 unmyelinated fibers. Note consistent representation of a marked number of fibers exhibiting a non-circular shape regardless of shape outcome measure used for analysis.

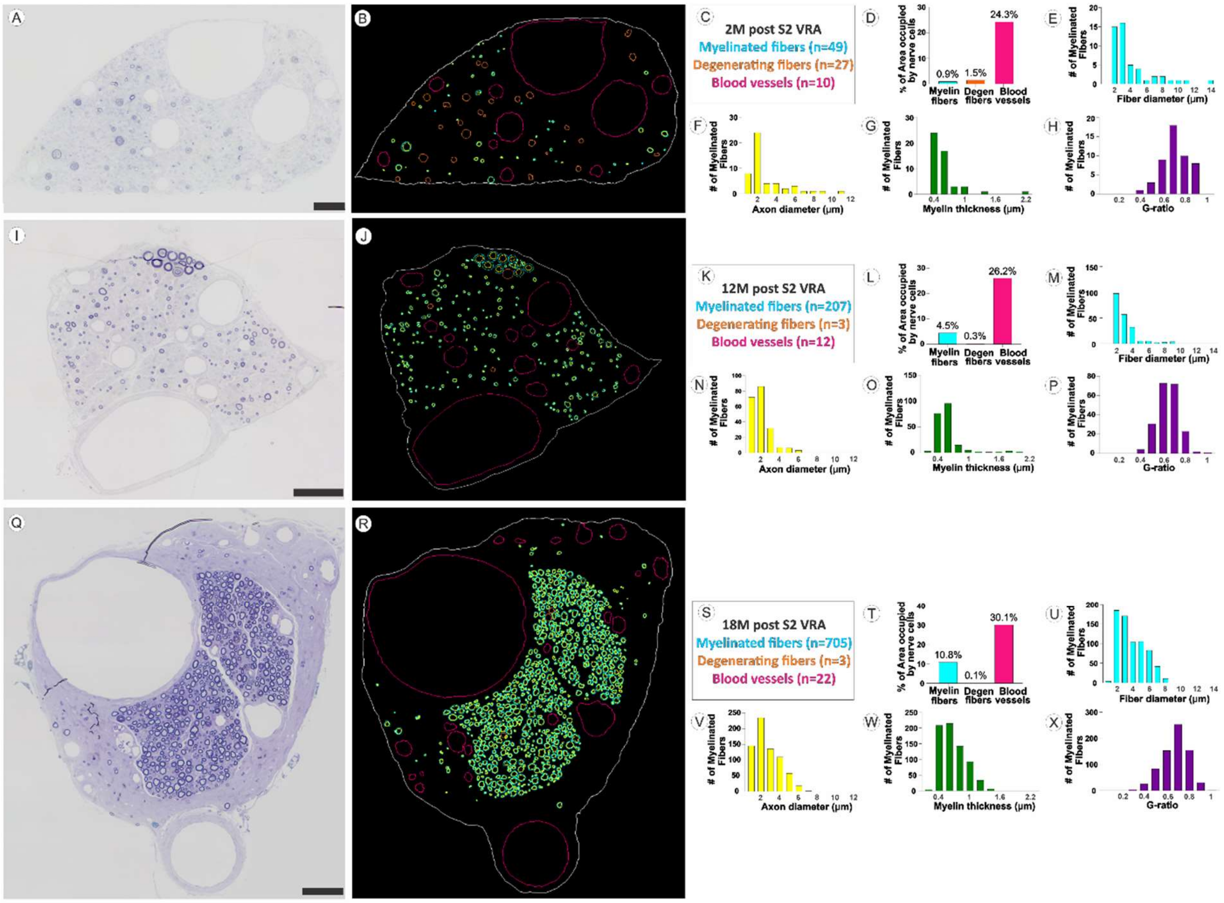

Supplementary Fig. S5. Morphometric analysis of regenerated and degenerating fibers in rhesus macaques at 2, 12, and 18 months post S2 ventral root avulsion using a SAE approach. A, I, Q. Light micrograph showing nerve root labeled with toluidine blue solution at 2, 12, and 18 months post injury respectively. **B, J, R.** Segmentation of myelinated fibers, degenerating fibers, blood vessels, and fascicle area at 2, 12, and 18 months post injury respectively. **C, K, S.** Summary of myelinated fibers, degenerating fibers, and blood vessels quantification at 2, 12, and 18 months post injury respectively. **D, L, T.** Bar graph showing the percentage of area occupied by nerve cells within the nerve root at 2, 12, and 18 months post injury respectively. **E-H, M-P, U-X.** Frequency distribution of myelinated fibers diameter, axon diameter, myelin thickness and G-ratio at 2, 12, and 18 months post injury respectively. Nerve cells were segmented in different colors for better identification and mapping; myelinated fiber

(light blue), myelinated axon (yellow), degenerating axon (orange), blood vessel (magenta), fascicle area (white). Scales: 50μm in **A**, **I**, and **Q**.
